# DIVERSITY, DISCOVERY, AND AGING REIMAGINED: NIA SESSION FOR EARLY-CAREER RESEARCHERS

**DOI:** 10.1093/geroni/igac059.805

**Published:** 2022-12-20

**Authors:** Melinda Kelley, Melinda Kelley

## Abstract

The National Institute on Aging (NIA) at the National Institutes of Health, Department of Health and Human Services, supports biomedical and behavioral research with a lifespan focus. NIA research seeks to understand the basic processes of aging, improve prevention and treatment of diseases in later life, and improve the health of older persons, in addition to a focus on Alzheimer’s disease and related dementias. NIA also supports the training and career development of scientists focusing on aging research and the development of research resources. This symposium, meant for junior faculty and emerging scholars, will provide an update on the latest research findings from NIA followed by a segment on funding mechanisms and strategies. An opportunity will be provided to meet and consult with NIA extramural staff.

